# Secondary succession of shrub-herb communities in the hilly area of Taihang Mountain

**DOI:** 10.3389/fpls.2023.1194083

**Published:** 2023-09-08

**Authors:** Xiuping Liu, Wangming Zhou, Xiaoxin Li, Yuming Zhang, Wenxu Dong

**Affiliations:** ^1^ Key Laboratory of Agricultural Water Resources, Hebei Key Laboratory of Soil Ecology, Center for Agricultural Resources Research, Institute of Genetics and Developmental Biology, Chinese Academy of Sciences, Shijiazhuang, China; ^2^ School of life Sciences, Anqing Normal University, Anqing, China

**Keywords:** *Vitex negundo* var. *heterophylla*, secondary succession, community structure, soil property, Taihang Mountain

## Abstract

**Introduction:**

To document the successional processes of shrub-herb communities after large-scale human disturbance, and understand how changing environmental conditions affect species replacement in semi-humid hilly areas.

**Methods:**

Utilizing the established permanent plots in the hilly area of Taihang Mountain, we evaluated temporal patterns of vegetation and soil following grass-to-shrub succession.

**Results and Discussion:**

Along secondary succession, *Vitex negundo* var. *heterophylla* gradually dominated in dry sunny slope and shared the dominance with *Leptodermis oblonga* in shaded slope. Herbaceous dominant species in shrub-herb communities switched from *Themeda japonica*, *Bothriochloa ischaemum*, *Artemisia sacrorum*, and *Cleistogenes chinensis* in 1986 census to *B*. *ischaemum* and *A*. *sacrorum* in 2008 census, but herb was no longer dominant in 2020 census. As succession progresses, species dominance increased while richness decreased generally, and herb cover and aboveground biomass decreased, whereas shrub height, cover, and aboveground biomass increased significantly. Soil organic matter (SOM), total nitrogen (TN), total phosphorus (TP), and total potassium (TK) in topsoil increased significantly while pH declined by 1.04 units over the past three decades. Plant communities transitioned from perennial herbs to shrub-herb and then shrub communities, and *V*. *negundo* var. *heterophylla* dominated in the succession of shrub-herb communities. Climate and soil properties, combined with plant attributes, together drive post-disturbance secondary succession. From a management perspective, the tight coupling between vegetation and soil under local climatic conditions should be considered to improve the fragile ecosystem in the hilly area of Taihang Mountain.

## 1 Introduction

Semi-humid ecosystems (precipitation 400-800 mm yr^-1^) cover about 6% of the global land area and provide crucial ecosystem services for human society (Calvo et al., 2002a; Wang et al., 2014). However, during several decades prior to the 1970s, large-scale human disturbances, such as deforestation, overgrazing, and excessive reclamation, have severely destroyed tree-dominated ecosystems and converted it into shrub-herb communities (Calvo et al., 2002b; Baudena et al., 2020). Even at deteriorated state, these shrub-herb communities provide at least partial ecosystem functions of primary forests and serve as biodiversity refuges, but their successional trajectories, especially in semi-humid hilly areas, depend on a variety of factors related to climate, topography, soils, and so on (Chua et al., 2013; Lai et al., 2020).

The successional trajectory of shrub-herb communities following disturbance is likely to vary widely across landscape, even among those with similar disturbance histories (Norden et al., 2015). Changing environmental conditions, such as temperature (Dodson and Root, 2013), precipitation (José Vidal-Macua et al., 2017; Liu et al., 2022), topography (Boukili and Chazdon, 2017), and soil properties (Engelbrecht et al., 2007; Liu et al., 2022), often influence community reassembly processes and alter its functions composition (Norden et al., 2015). For semi-humid areas, expected increase in drought may promotes leaf abscission (Nepstad et al., 2007), retards plant growth (José Vidal-Macua et al., 2017), and potentially shifts community composition over time (Baudena et al., 2020; Liu et al., 2022). In particular, severe drought combined with high temperature can potentially impede vegetation post-recovery via alterations in growth pattern (Dodson and Root, 2013; Liu et al., 2022), or even lead to the eventual replacement of established forests by drought-tolerant shrubs communities (José Vidal-Macua et al., 2017; Baudena et al., 2020). In addition, topographic heterogeneity can drive shifts in soil microclimate environments, which may potentially affect community structure, composition, and dynamics (Das et al., 2015; Sanaphre-Villanueva et al., 2016; José Vidal-Macua et al., 2017; Jucker et al., 2018). Plant response to these environmental fluctuations generally varies among climatic regions, functional groups, and ecosystems (Chang and Turner, 2019), so accurately understanding vegetation successional processes will assist in disentangling the mechanisms that drive variability in these ecosystems, and enhance community resilience to climatic change.

The Taihang Mountains run from north to south in northern China, and form a natural boundary between Loess Plateau and North China Plain (Yang et al., 2006). The original vegetation of the region was of broadleaved deciduous forest, but large-scale deforestation (e.g., cutting, tilling, logging, grazing, and so on) before the 1970s has severely destroyed and turned it into degraded shrub-herb community (Pérez-Devesa et al., 2008; Liu et al., 2012; Sun et al., 2014). Starting from 1980s, a series of vegetation restoration projects, such as afforestation, banning grazing, and returning slope cropland forest or grassland, were implemented to control soil erosion, reduce ecological degradation, and promote vegetation recovery (Liu et al., 2014; Guo et al., 2021). In addition, annual mean air temperature across the study area exhibited a gradually increasing trend (Liu et al., 2023). Therefore, studies on species composition and community structure of shrublands on time-scales can help explore plant defense responses to natural stresses, predict the potential direction of vegetation succession, and inform appropriate environmental management strategies (Xie and Tang, 2017).

To contribute to a better understanding the succession processes of shrub-herb communities under climate changes, we established permanent plots in the hilly area of Taihang Mountain, China, and monitored vegetation changes in relation to various environmental conditions. Vegetation and soil census was conducted in 1986 followed by a re-census in 2008 and again in 2020 to address the following issues: (1) quantify how vegetation and soil shifts during secondary succession; (2) examine which factors are closely related to the changes in community structure; and (3) elucidate the physio-morphological adaptations of *Vitex negundo* var. *heterophylla* to environmental changes during vegetation succession.

## 2 Materials and methods

### 2.1 Site description

This study was conducted in Niujiazhuang Catchment of middle Taihang Mountain, China (114°15′50″ E, 37°52′44″ N). Elevation ranges from 247 to 1040 m a.s.l. and slope varies from 20 to 45°. The climate is temperate continental monsoon, with warm summers and cool winters. Annual precipitation (1962-2020) averages 519 mm, ranging from 200 to 1129 mm, with 74% falling between June and September (Liu et al., 2023). The monthly mean air temperature (1962-2020) was -3.07°C in January and 26.8°C in July (Liu et al., 2023). The study site had a total area of 9.3 km^2^ and was located in an area of lowland hills with a relatively steep slope.

The soil parent materials in the area are mainly granite, granite porphyry, limestone, sandstone, and shale. Soils in the area are highly-weathered mountainous cinnamon soils (classified as Ustalf), mainly derived from gneissic granitoid, and partly come from limestone and shale (Liu et al., 2014). These soils are deep and well drained on north slopes but skeletal on south slopes, with limited organic matter and low water holding capacity.

Vegetation in the area is a mosaic of shrubs, herbs, plantation, deciduous and coniferous forests, and agricultural crops. Shrub-herb community originated by natural regeneration after the destruction of original forest vegetation, of which the dominant species *Leptodermis oblonga* is accustomed to growing in shaded slope, whereas *V*. *negundo* var. *heterophylla* and *Ziziphus jujuba* var. *spinosa* prefer dry sunny slope (Liu et al., 2014). As affected by climate related environmental change, *V*. *negundo* var. *heterophylla* gradually spread northward to occupy available ecological niches for *L*. *oblonga*. Natural grasslands are commonly found on gentle north-facing slopes, after 30 years of natural regeneration, grassland in the area has undergone species replacement, such as *Bothriochloa ischaemum* and *Artemisia sacrorum* are still dominant today, while *Themeda japonica* and *Cleistogenes chinensis* occur sporadically, but *Pennisetum centrasiaticum* and *Achnatherum extremiorientale* were no longer present in 2020 census. *Robinia pseudoacacia* used to be a common tree species in afforestation programs, and was widely planted on Taihang Mountain from 1980s, but now are sparsely distributed in this area and experienced extensive invasion of *V*. *negundo* var. *heterophylla* through natural succession (Liu et al., 2014).

### 2.2 Field study

In 1986, 144 2 m × 2 m permanent plots were established along 42 30-m long transects (parallel to the slope) to monitor the post-disturbance succession processes of shrub-herb communities. These plots are stratified to capture changes in aspect, slope position, and soil type across the sites and to obtain a reasonable representation of the entire range (valley to ridge). Within each of these plots, we conducted vegetation and soil census in both 1986 and 2008, and in 2020, all plots recorded were grouped by vegetation type and geographic location and then were classified into *V*. *negundo* var. *heterophylla* shrubland (VS), *L*. *oblonga* shrubland (LS), *R*. *pseudoacacia* plantation (RP), and grassland (G). Finally, a total of 34 plots (8 VS, 8 LS, 8 RP, and 10 G) with expanded area up to 16 m^2^ were selected for re-census.

In each plot, the height, cover, and number of each shrub or herb species, and cover and aboveground biomass of all shrubs or herbs were recorded, and topographical factors such as elevation, slope gradient, slope aspect, and slope position, were also recorded for each sampling plot. The aboveground biomass of shrubs and herbs were collected within neighboring areas of each plot. The plant samples were oven-dried at 80°C for at least 72 hours to a constant weight, and then dry matter were weighed to determine total biomass. After biomass harvesting, three soil samples (0-20 cm) in each plot were collected along the transect of neighboring areas using a 5 cm diameter soil auger, all visible soil organisms, stones, and plant debris were removed, and the soils were sieved using a 2 mm sieve before laboratory analysis.

### 2.3 Soil chemical properties

Soil pH was measured in a soil/water ratio of 1:2.5 using a pH meter (Bao, 2000), soil organic matter (SOM) was analyzed using the Walkley-Black method (Nelson and Sommers, 1996), total nitrogen (TN) was determined by the Kjeldahl method (Bremner, 1996), total phosphorus (TP) was digested by perchloric acid and determined by the molybdate colorimetric method (O’Halloran and Cade-Menun, 2006), and total potassium (TK) were analyzed by flame atomic absorption spectrophotometer.

### 2.4 Data analysis

To account for the variation in community structure in terms of vegetation type and environmental conditions, all data collected were classified into four groups (VS, LS, RP, and G), and the differences in community compositions and soil properties were compared among plots and across the three census period.

In each plot, the importance value (IV) was calculated as the sum of relative cover, relative height, and relative frequency for herbs and shrubs (Do et al., 2019). Shannon-Wiener diversity index (H, H=-Σ*p_i_* ln*p_i_*, *p_i_* is the proportion of individuals found in species *i*), species richness index (S, the number of species), and Simpson’s dominance index (D, D = 1–Σ*p_i_* ^2^) were used to assess the changes in species diversity during succession.

Analysis of variance (ANOVA) and least significant difference (LSD) multiple range tested at an alpha-level of 0.05 were used to compare the difference in species diversity (S, D, and H), community structure (height, cover, and aboveground biomass), and soil properties (SOM, TN, TP, and TK) for the entire 0-20 cm soil layer across plots for census interval.

There is only one weather station in the study area and we could not obtain meteorological data for each plot, we used the equations of Guo (1994) and Yang et al. (2006) for the Taihang Mountains to estimate precipitation and temperature at each sampling plot.


(1) $$\text{P}=519.23+151.62\times (\text{E}/1000)-43.26\times {\left(\text{E}/1000\right)}^{2}$$

(2) T=15.4−0.628×(L−34.7)−0.522×(E/100)

where P (mm) is precipitation, E (m) is elevation, T (°C) is annual average air temperature, L (°) is latitude. Since the study area is a small watershed, the latitudinal differences between sampling plots and weather station are small, we ignored the influence of latitude on temperature.

Multivariate analysis was performed through CANOCO 5 to identify the relationship between environmental variables (climate, topography, and soil properties) and vegetation growth, and examine which factors are closely related to the changes in community structure. At first, detrended correspondence analysis (DCA) was conducted to determine whether to use linear or unimodal numerical methods, and the length of the first DCA ordination axis we obtained was lower than 3 SD (**Table 1**), indicating that linear model with redundancy analysis (RDA) was the most appropriate ordination method for direct gradient analysis. Accordingly, a final set of nine environmental (precipitation, temperature, slope aspect, slope position, pH, SOM, TN, TP, and TK) and nine vegetation variables (S, D, H, shrub height, shrub cover, shrub biomass, herb height, herb cover, and herb biomass) were selected to construct RDA analysis.

**Table 1:** Redundancy analysis of shrubland from 1986 to 2008.

Axes	1	2	3	4
Lengths of gradient (Checked by DCA)	1.50	0.57	0.31	0.37
RDA				
Eigenvalues	0.2238	0.0306	0.0226	0.0078
Explained variation (cumulative)	22.38	25.44	27.69	28.47
Pseudo-canonical correlation	0.6918	0.5184	0.6783	0.3784
Explained fitted variation (cumulative)	77.46	88.06	95.87	98.57

## 3 Results

### 3.1 Species diversity

**Figure 1:**
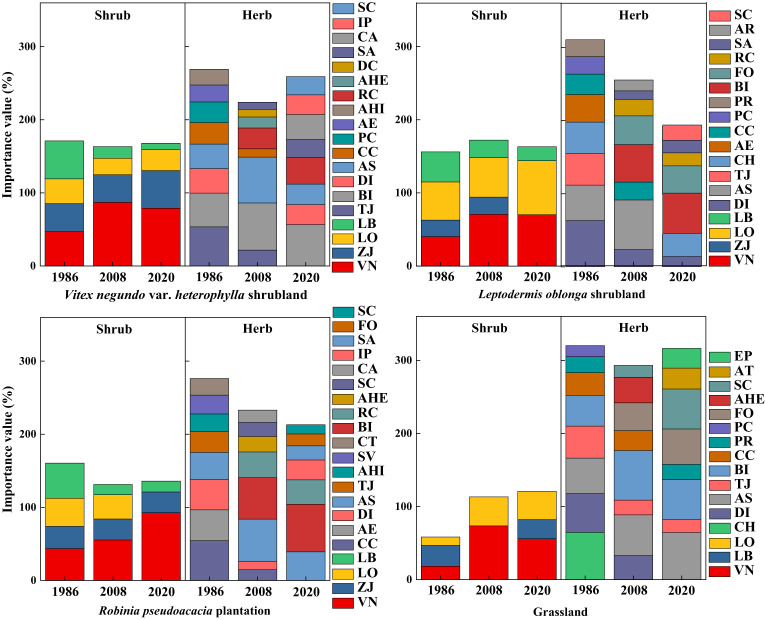
Changes in importance values of major species with secondary succession. AE, *Achnatherum extremiorientale*; AHI, *Arthraxon hispidus*; AHE, *Artemisia hedinii*; AR, *Allium ramosum*; AS, *Artemisia sacrorum*; AT, *Adenophora tetraphylla*; BI, *Bothriochloa ischaemum*; CA, *Chenopodium album*; CC, *Cleistogenes chinensis*; CH, *Carex humilis*; CT, *Cynanchum thesioides*; DC, *Dianthus chinensis*; DI, *Dendranthema indicum*; EP, *Euphorbia pekinensis*; FO, *Festuca ovina*; IP, *Ixeris polycephala*; LB, *Lespedeza bicolor*; LO, *Leptodermis oblonga*; PC, *Pennisetum centrasiaticum*; PR, *Patrinia rupestris*; RC, *Rubia cordifolia*; SA, *Scorzonera austriaca*; SC, *Salsola collina*; SV, *Setaria viridis*; TJ, *Themeda japonica*; VN, *Vitex negundo var. heterophylla*; ZJ, *Ziziphus jujuba var. spinosa*.

Along secondary succession, *V*. *negundo* var. *heterophylla* was the species with greatest important value in VS, RP, and G, and shared the dominance with *L*. *oblonga* in LS (**Figure 1**). Herbaceous dominant species in shrub-herb communities switched from *T*. *japonica*, *B*. *ischaemum*, *A*. *sacrorum*, and *C*. *chinensis* in 1986 census to *B*. *ischaemum* and *A*. *sacrorum* in 2008 census, but herb was no longer dominant in 2020 census (**Figure 1**).

**Figure 2:**
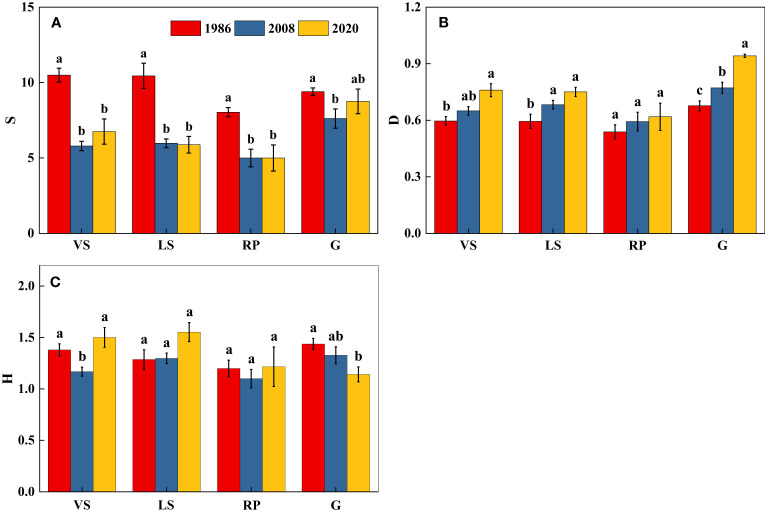
Changes in species diversity of shrub-herb communities with succession. Different letters indicate significant differences between census years at *p*< 0.05 according to ANOVA. **(A)** Species richness index (S); **(B)** Simpson’s dominance index (D); **(C)** Shannon-Wiener’s index (H); VS, *Vitex negundo* var. *heterophylla* shrubland; LS, *Leptodermis oblonga* shrubland; RP, *Robinia pseudoacacia* plantation, G, grassland.

There was no significant difference in diversity (H) of VS, RP, and G among three census periods (*p* > 0.05) (**Figure 2**). Species richness (S) generally decreased while dominance (D) increased over the 34-year time period (*p*< 0.05) (**Figure 2**).

### 3.2 Community structure

**Figure 3:**
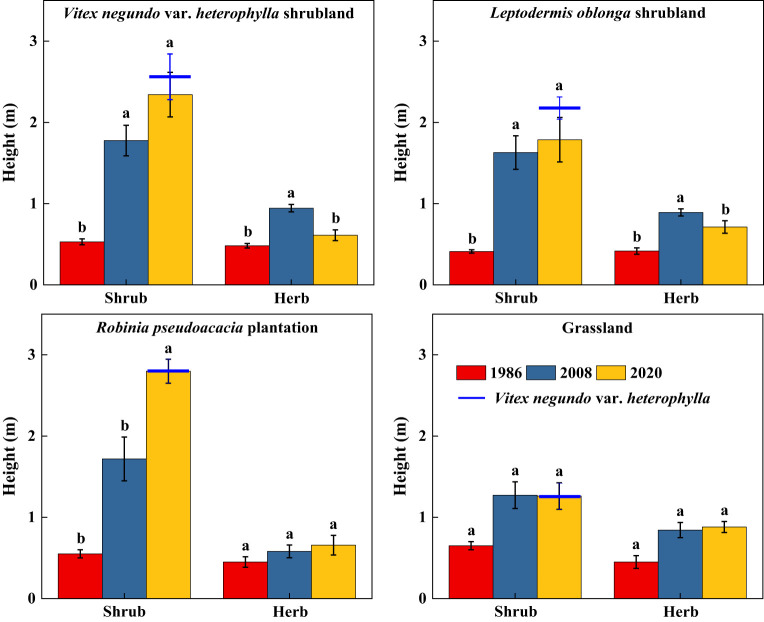
Changes in height of shrub-herb communities with succession. Different letters indicate significant differences between census years at *p*< 0.05 according to ANOVA.

**Figure 4:**
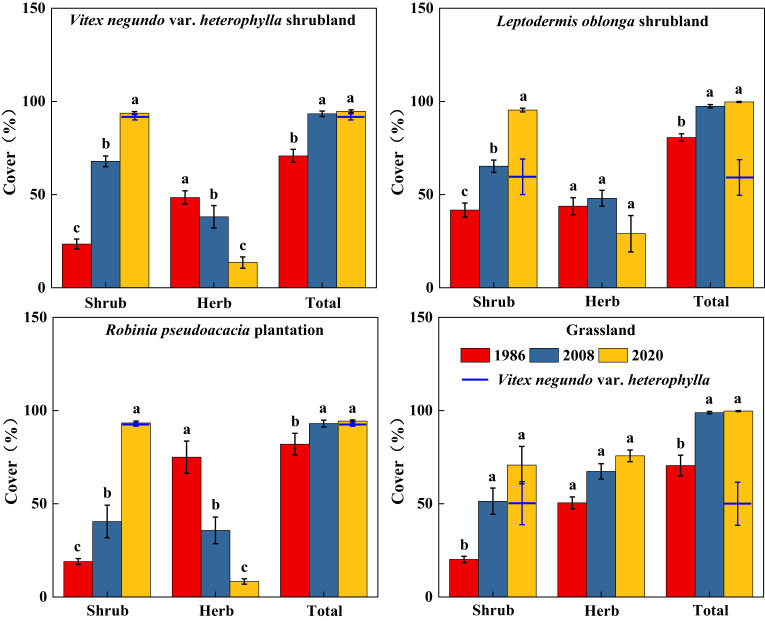
Changes in cover of shrub-herb communities with succession. Different letters indicate significant differences between census years at *p*< 0.05 according to ANOVA.

**Figure 5:**
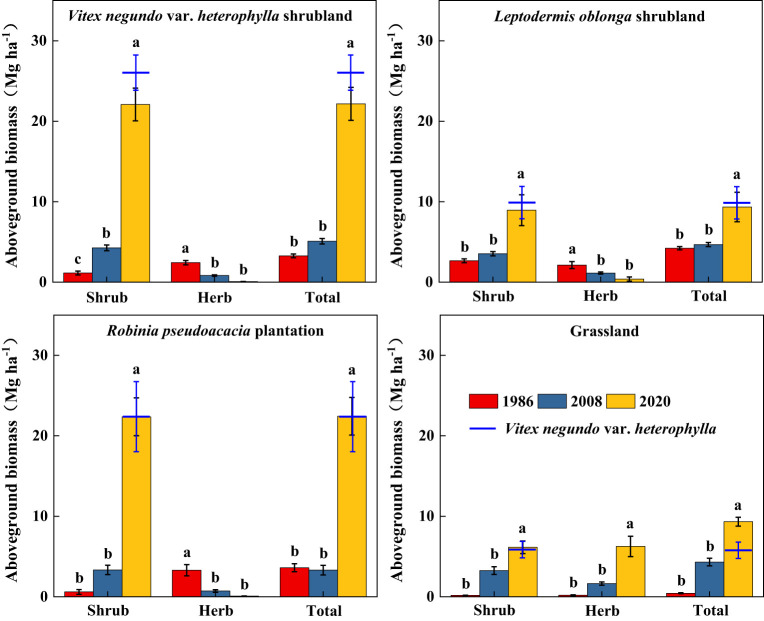
Changes in aboveground biomass of shrub-herb communities with succession. Different letters indicate significant differences between census years at *p*< 0.05 according to ANOVA.

Along secondary succession, shrub height in VS, LS, and RP significantly increased (*p*< 0.05), whereas herb height remained stable (**Figure 3**). Shrub cover significantly increased after 34 years of vegetation recovery, whereas herb cover in VS and RP significantly decreased (*p*< 0.05) (**Figure 4**). Shrub aboveground biomass significantly increased with succession, whereas herb aboveground biomass in VS, LS, and RP significantly decreased (*p*< 0.05) (**Figure 5**). Plant communities transitioned from perennial herbs to shrub-herb and then shrub communities.

In 2020, community mean height, cover, and aboveground biomass were 2.19m, 97.2%, and 15.7 Mg ha^-1^, whereas the values of *V*. *negundo* var. *heterophylla* were 2.37m, 78.0%, and 17.9 Mg ha^-1^, respectively (**Figures 3**–**5**). *V*. *negundo* var. *heterophylla* was the most dominant species in the hilly area of Taihang Mountain.

### 3.3 Soil property

**Figure 6:**
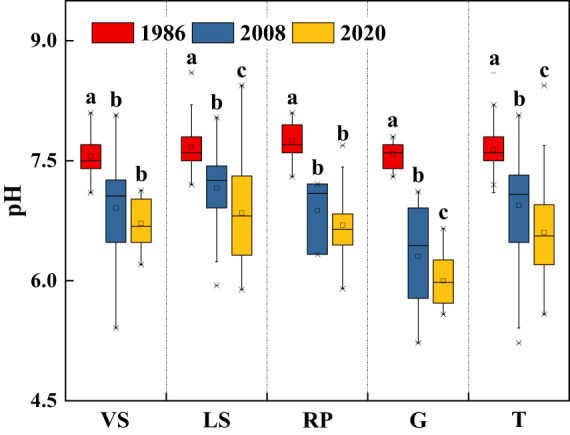
Changes in soil pH in topsoil (0-20 cm) of shrub-herb communities with succession. Different letters indicate significant differences between census years at *p*< 0.05 according to ANOVA. VS, *Vitex negundo* var. *heterophylla* shrubland; LS, *Leptodermis oblonga* shrubland; RP, *Robinia pseudoacacia* plantation; G, Grassland; T, Total.

**Figure 7:**
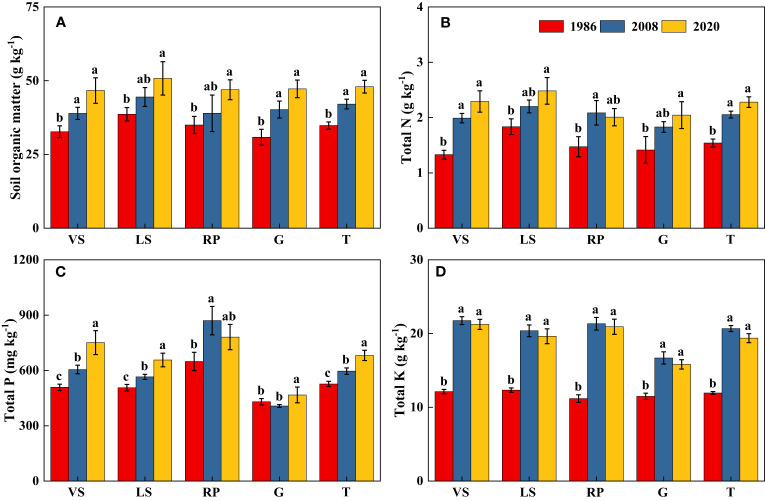
Changes in soil organic matter **(A)**, total N **(B)**, total P **(C)**, and total K **(D)** in topsoil (0-20 cm) of shrub-herb communities with succession. Different letters indicate significant differences between census years at *p*< 0.05 according to ANOVA. VS, *Vitex negundo* var. *heterophylla* shrubland; LS, *Leptodermis oblonga* shrubland; RP, *Robinia pseudoacacia* plantation; G, Grassland; T, Total.

Soil pH in topsoil declined significantly over the past three decades (*p*< 0.05), from 7.64 in 1986 to 6.60 in 2020, with a net decrease of about 1.04 units (**Figure 6**). Long-term secondary succession increased SOM content in topsoil, on average, by 37.0% (**Figure 7**). From 1986 to 2020, Surface TN and TP contents in VS, LS, and G increased significantly (*p*< 0.05), while in RP, the changes tended to be gradual (**Figure 7**). Surface TK content increased quickly during the first 20 years of vegetation restoration and tended to be stable thereafter (**Figure 7**).

### 3.4 Effects of environmental variables

**Figure 8:**
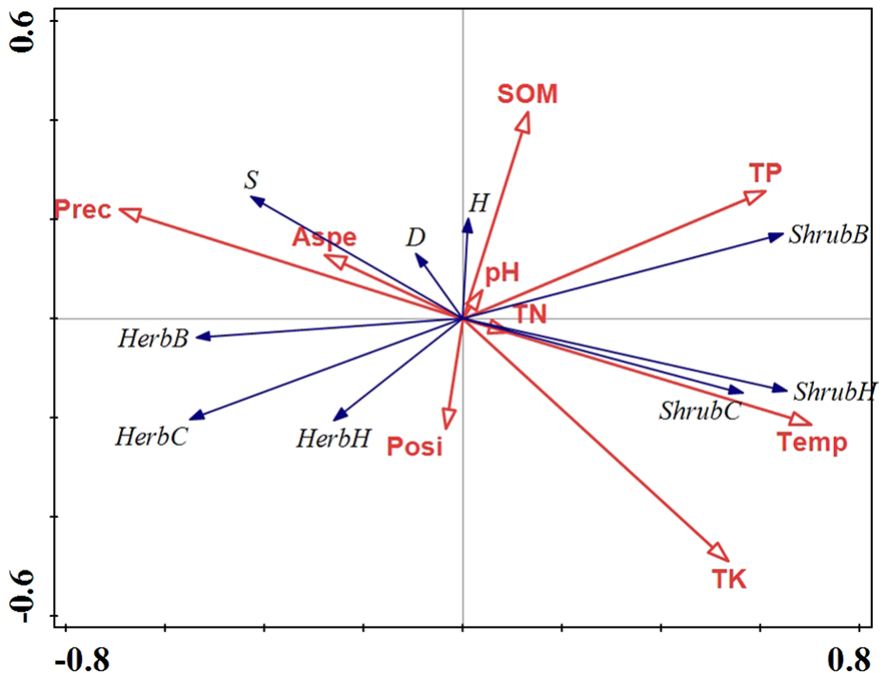
Redundancy analysis (RDA) of environmental and vegetation variables from 1986 to 2020. Prec, precipitation; Temp, temperature; Aspe, slope aspect; Posi, slope position; SOM, soil organic matter; TN, total N; TP, total P; TK, total K; S, species richness index; D, Simpson’s dominance index; H, Shannon-Wiener’s index; ShrH, shrub height; ShrC, shrub cover; ShrB, shrub biomass; HerH, herb height; HerC, herb cover; HerB, herb biomass.

RDA analysis showed that there was a strong correlation between vegetation and environmental variables, with pseudo-canonical correlation of 0.692 on the first axis and 0.518 on the second axis (**Table 1**). The cumulative percentage variance of vegetation-environment relations of the first and second axis were 77.5 (eigenvalue 0.224) and 88.1% (eigenvalue 0.031), respectively (**Table 1**). The RDA results also indicated that temperature, TP, TK, and precipitation were the most important factors for community structure, contributing respectively 39.1 (p<0.01), 18.9 (p<0.01), 8.3 (p<0.05), and 7.7% (p<0.05) of the total variation (**Figure 8**, **Table 2**).

**Table 2:** Summary of the forward selection procedure in the redundancy analysis (RDA).

Name	Explains (%)	Contribution (%)	F	P
Temp	11.3	39.1	9.3	0.002
TP	5.5	18.9	4.7	0.002
TK	2.4	8.3	2.1	0.042
Prec	2.2	7.7	2.0	0.046
pH	2.1	7.2	1.9	0.050
Aspe	1.7	5.9	1.6	0.094
TN	1.5	5.3	1.4	0.094
Posi	1.4	5.0	1.3	0.110
SOM	0.7	2.5	0.7	0.436

Prec, precipitation; Temp, temperature; Aspe, slope aspect; Posi, slope position; SOM, soil organic matter; TN, total N; TP, total P; TK, total K.

## 4 Discussion

*V*. *negundo* var. *heterophylla* is a highly water-consuming plant (Si et al., 2020), how did it become the most dominant species in semi-humid climate zone? Wang et al. (2017) and Zhu et al. (2016) reported that *V*. *negundo* var. *heterophylla* possesses a functionally dimorphic root system, which allows it to use both shallow and deep soil water, or even ground water (Moreira et al., 2003; Damascos et al., 2005). In addition, Du et al. (2010) and Zhang (2007) pointed out the sprouting time of *V*. *negundo* var. *heterophylla* could vary with environmental conditions, they sprouted new leaves in mid-April at mild-moderate drought while in early May at severe drought, but almost stagnant growth at extreme drought. Notably, Yan et al. (2000) found that the numerous epidermal hairs of *V*. *negundo* var. *heterophylla* could shielded most stomata and formed a relatively independent system for reducing water transpiration. Therefore, the high degree of morphological plasticity, conservative light utilization strategy, and strong recovery ability after disturbance make *V*. *negundo* var. *heterophylla* a widely predominant species in warm-temperate regions (Li et al., 2017).

Aboveground biomass accumulation increased during secondary succession (**Figure 5**) (Castro and Freitas, 2009; Enes et al., 2020; Ma and Wang, 2020). Averaged over all plots, the aboveground biomass of shrubland increased over 5 times (**Figure 5**), similar to the results of Yang et al. (2017) and Petrie et al. (2015). Despite differences in vegetation type, secondary shrublands following disturbance are substantial carbon sinks and that this capacity to store carbon increases with succession (Adhikari and White, 2016; Kong et al., 2022). In addition, previous studies reported species-rich stands had higher carbon stocks than stands with low richness (Chen et al., 2018; Liu et al., 2018), and Chen et al. (2018) proposed high plant diversity increased above- and belowground biomass as well as the resistance of productivity to climate extremes. While, in our study, the relationship between aboveground biomass and species diversity weakened with succession (**Figures 2** and **5**), Potter and Woodall (2014) and Marin-Spiotta et al. (2007) also obtained similar results. This demonstrated that a limited number of species well-adapted to the local conditions promoted vegetation recovery and accumulated biomass in semi-humid areas (El-Sheikh, 2005; Potter and Woodall, 2014).

Soil pH in the hilly area of Taihang Mountain declined significantly over the past three decades, with an overall decrease of 1.04 units, similar to those reported by Yang et al. (2012) and Zhang et al. (2022) for China’s soils. Soil acidification is the result of atmospheric deposition, plant growth, and soil forming processes (Ritter et al., 2003; De Schrijver et al., 2006; Hong et al., 2018). Undoubtedly, atmospheric deposition reduced soil pH (De Schrijver et al., 2012; Yang et al., 2015). Whereas, plant metabolism could neutralize soil pH (Ovington, 1953; De Schrijver et al., 2012; Chen et al., 2019), and the minerals/ions released from granite and gneiss through weathering could buffer soil acidification (Rhoades and Binkley, 1996; Drohan and Sharpe, 1997). However, soil pH in our study area declined significantly. Limited precipitation may contribute to the absence of soil animals and microorganisms, which retard litter decomposition and mineral weathering, and consequently result in soil acidification (Shu et al., 2019).

Despite the relatively decrease of soil pH, soil nutrients in topsoil accumulated gradually as succession progresses. We found SOM content increased on average by 37% from 1986 to 2020 (**Figure 7**), Piche and Kelting (2015) and Sokolowska et al. (2020) obtained similar results. This suggested that organic matter inputs exceeded decomposition outputs over time of secondary succession (Nadal-Romero et al., 2016; Sokolowska et al., 2020). As with SOM, TN content in topsoil significantly increased, similar to the observations of Deng et al. (2014) and Segura et al. (2020). The apparent increase in topsoil TN could be attributed to the release of N from plant residues (Johnson et al., 2016), the return of N from subsoil (Smal et al., 2019), and atmospheric N deposition (Falkengren-Grerup et al., 2006). Similar to SOM and TN, TP and TK contents in the top 20 cm of soil increased progressively with shrub age, this agreed with the results of Du et al. (2007). The commonly observed increase in topsoil P may be achieved by atmospheric P deposition (Cao et al., 2011) and P translocation from depth in the soil profile to the surface soil (Sullivan et al., 2019). Similarly, high levels of K in the 0-20 cm soil may come from the K uptake by roots from deeper soil layers to the topsoil (Segura et al., 2020). Consequently, vegetation succession in the hilly area of Taihang Mountain significantly increased most of the measured soil parameters, and consequently improved soil quality.

Climate varied with altitude results in different vegetation and soil, and soil, in turn, influences plant growth (Miki and Kondoh, 2002; Du et al., 2014). Among the environmental variables, temperature, TP, TK, and precipitation were important factors in explaining variations in community structure of Taihang Mountain. Several reports indicated that precipitation and temperature were the most important factors influencing plant growth, community structure and function (Yang et al., 2006; Nunes et al., 2019; Zhou et al., 2019). For example, Zhou and Zhang (1996) reported that widespread drought driven by high temperatures or low precipitations could lead to substantial forest decline, or replacement of drought-vulnerable with drought-tolerant species (Falk et al., 2019). As temperature rise and water availability decreases (Liu et al., 2023), the more drought-tolerant *V*. *negundo* var. *heterophylla* becomes the most dominant species in the hilly area of Taihang Mountain. As expected, vegetation recovery generally improved soil quality, in turn, soil properties also determine plant community composition (Zhang et al., 2006; Zhao et al., 2017). The increased soil nutrients, in particular P and K, could facilitate shrub vegetation restoration in this region. Thus, to improve and restore the fragile ecological ecosystem in the hilly area of Taihang Mountain, we should consider the tight coupling between vegetation and soil under local climatic conditions.

## 5 Conclusion

Along secondary succession, *V*. *negundo* var. *heterophylla* gradually dominated in dry sunny slope and shared the dominance with *L*. *oblonga* in shaded slope. Herbaceous dominant species in shrub-herb communities switched from *T*. *japonica*, *B*. *ischaemum*, *A*. *sacrorum*, and *C*. *chinensis* in 1986 census to *B*. *ischaemum* and *A*. *sacrorum* in 2008 census, but herb was no longer dominant in 2020 census. As succession progresses, species dominance increased while richness decreased generally, and herb cover and aboveground biomass decreased, whereas shrub height, cover, and aboveground biomass increased significantly. Long-term secondary succession increased topsoil SOM, TN, TP, and TK significantly while declined pH by 1.04 units. Plant communities transitioned from perennial herbs to shrub-herb and then shrub communities, and *V*. *negundo* var. *heterophylla* dominated in succession of shrub-herb communities. Climate and soil properties, combined with plant attributes, together drive vegetation post-recovery.

## Data availability statement

The raw data supporting the conclusions of this article will be made available by the authors, without undue reservation.

## Author contributions

XPL and WZ: Investigation, Formal analysis, Writing - Original Draft; XXL and YZ: Methodology, Data Curation, Writing - Review & Editing; WD: Resources, Supervision, Project administration. All authors contributed to the article and approved the submitted version.
